# Calmodulin levels in blood cells as a potential biomarker of Alzheimer’s disease

**DOI:** 10.1186/alzrt219

**Published:** 2013-11-07

**Authors:** Noemí Esteras, Carolina Alquézar, Ana de la Encarnación, Alberto Villarejo, Félix Bermejo-Pareja, Ángeles Martín-Requero

**Affiliations:** 1 Department of Cellular and Molecular Medicine, Centro de Investigaciones Biológicas (CSIC), Ramiro de Maeztu 9, 28040 Madrid, Spain; 2 Centro de Investigación Biomédica en Red de Enfermedades Raras (CIBERER), Valencia, Spain; 3 Department of Neurology, Hospital Doce de Octubre, Avda de Córdoba s/n, 28041 Valencia, Spain; 4 Centro de Investigación Biomédica en Red de Enfermedades Neurodegenerativas (CIBERNED), Madrid, Spain

## Abstract

**Introduction:**

The clinical features of Alzheimer’s disease (AD) overlap with a number of other dementias and conclusive diagnosis is only achieved at autopsy. Accurate in-life diagnosis requires finding biomarkers suitable for early diagnosis, as well as for discrimination from other types of dementia. Mounting evidence suggests that AD-dependent processes may also affect peripheral cells. We previously reported that calmodulin (CaM) signaling is impaired in AD lymphoblasts. Here, we address the issue as to whether the assessment of CaM levels in peripheral cells could serve as a diagnostic biomarker.

**Methods:**

A total of 165 subjects were enrolled in the study, including 56 AD patients, 15 patients with mild cognitive impairment, 7 with frontotemporal dementia associated with *progranulin* mutations, 4 with dementia with Lewy bodies, 20 patients with Parkinson’s disease, 10 with amyotrophic lateral sclerosis, 5 with progressive supranuclear palsy, and 48 cognitively normal individuals. CaM levels were then analyzed in lymphoblasts, peripheral blood mononuclear cells and plasma. Receiver operating characteristic (ROC) curve analyses were employed to evaluate the diagnostic performance of CaM content in identifying AD patients.

**Results:**

Compared with control individuals, CaM levels were significantly increased in AD cells, but not in the other neurodegenerative disorders. CaM levels differentiated AD from control with a sensitivity of 0.89 and a specificity of 0.82 and were not dependent on disease severity or age. MCI patients also showed higher levels of the protein.

**Conclusions:**

CaM levels could be considered a peripheral biomarker for AD in its early stage and help to discriminate from other types of dementia.

## Introduction

Alzheimer’s disease (AD) is the most common form of dementia in older people. The diagnosis of AD is made following clinical criteria and only postmortem autopsy can really confirm the disease [1,2]. AD diagnosis is time consuming and requires a combination of clinical assessment, psychological testing, imaging and exclusion of other neurological disorders. The availability of reliable minimally invasive biomarkers for AD progression, and especially for incipient AD, would be of great interest for an early diagnosis and hopefully to slow disease progression.

AD pathogenesis is not completely understood and diagnosis often occurs after significant neuronal loss and pathology have occurred. However, a variety of postmortem evidence suggests that the pathological hallmarks of AD begin to occur early in an individual’s life. Both disease-specific genetics and environmental factors affect cellular pathways before the clinical onset. Alterations in cellular response to these stressors are not restricted to neurons, representing a systemic pathophysiological process [3-7]. For example, the accumulation of senile plaques in the central nervous system formed by amyloid-beta deposits is the main hallmark of the disease [8], but is also present in the periphery and can be detected in blood [9]. Moreover, systemic changes at the immunological level have been reported to be associated with increased inflammation in the brain [10,11], indicating that the hematopoietic system and the central nervous system are affected similarly by AD-dependent processes. Blood cells, easily accessible from patients, may therefore display disease-specific signature molecules that eventually could serve as biomarkers for AD. A blood-based biomarker would be ideal since venous puncture is a widely accepted procedure with no risk and low cost for sample collection.

Work carried out in our laboratory in the past few years aimed at investigating whether changes in cell cycle-related events could be important in the neurodegenerative process, and to demonstrate the usefulness of peripheral cells from AD patients to determine AD pathogenesis [7,12]. We reported a functional relationship between Ca^2+^/calmodulin (CaM) and the main signaling pathways controlling cell survival or death depending upon growth factor availability in either Epstein–Barr virus-immortalized lymphocytes or freshly isolated lymphocytes [13-15]. These features were considered peripheral signs of the disease, because current evidence relates the process of neuronal apoptosis occurring in AD to the aberrant re-entry of differentiated neurons into the cell cycle [16-18]. Moreover, we detected significantly increased levels of CaM in AD lymphoblasts [19].

The present work was undertaken to elucidate whether higher CaM content is a characteristic feature of AD or whether it may occur in other neurodegenerative disorders. To this end, we determined CaM levels in lymphoblastoid cell lines and peripheral blood mononuclear cells (PBMCs) from nondemented individuals, and from subjects with mild, moderate or severe AD. A small group of mild cognitive impairment (MCI) individuals has been included. MCI is a prodromal phase of AD characterized by the onset of the earliest cognitive symptoms (typically deficits in episodic memory) that do not meet the criteria for dementia [20]. The time and sequence of changes in AD are considered important factors for improving early diagnosis and treatment [21,22]. In addition, we included samples from frontotemporal dementia (FTD) patients, carriers of the splicing mutation c.709-1G > A in the progranulin (*PGRN*) gene [23], patients with dementia with Lewy bodies (DLB), as well as patients suffering from Parkinson’s disease (PD), amyotrophic lateral sclerosis (ALS) and progressive supranuclear palsy (PSP) as other neurodegenerative disorders. We found that assessing the CaM content in lymphocytes could help to discriminate between AD patients and non-AD individuals including patients with other dementias or neurodegenerative disorders, and therefore the CaM content of peripheral cells may become a potential biomarker for AD.

## Methods

### Subjects

Healthy controls and patients enrolled in the study were recruited from Hospital Doce de Octubre, Madrid, Spain. Individuals carrying a loss-of-function *PGRN* mutation, c.709-1G > A [23], suffering from FTD were recruited in Hospital Donostia, San Sebastián, Spain. The clinical diagnosis of probable AD was based on the criteria of the National Institute of Neurological and Communicative Disorders and Stroke and the Alzheimer’s Disease and Related Disorders Association [1] and on the Diagnostic and Statistical Manual of Mental Disorders IV criteria, and diagnosis required evidence of cognitive decline (neuropsychological test battery, clinical mental examination) as well as evidence of impairment in social or occupational function. The Mini-Mental State Examination was used to assess cognitive function [24]. Classification of mild, moderate and severe degrees of AD was performed using Diagnostic and Statistical Manual of Mental Disorders III-R criteria. MCI was diagnosed using consensus criteria of the International Working Group on MCI [25]. The diagnosis of FTD was performed by applying consensus criteria as published elsewhere [26]. These individuals carry a loss-of-function *PGRN* mutation, c.709-1G > A, described previously [23]. Established criteria were applied for the diagnosis of DLB [27], ALS [28], probable PD [29] and PSP [30]. The control group was formed by individuals – in general, family members of the patients – with no signs of neurological disease or cognitive decline.

### Blood collection

This study was approved by the Ethic Committee of Clinical Investigation of Hospital 12 de Octubre and Hospital Donostia and by the Spanish Council of Higher Research Institutional Review Board. Informed consent from all subjects was obtained prior to their participation. Sampling protocols were similar in both clinical centers. Blood samples (approximately 8 ml) were obtained through antecubital vein puncture in ethylenediamine tetraacetic acid-treated Vacutainer® tubes (BD, Madrid, Spain). Plasma was obtained after centrifugation (2,000 rpm, 10 minutes), apportioned into 500 μl aliquots in polypropylene tubes, and stored at −80°C.

### Isolation of peripheral blood mononuclear cells and establishment of lymphoblastoid cell lines

PBMCs were isolated on Lymphoprep™ density-gradient centrifugation according to the instructions of the manufacturer (Axix-Shield Po CAS, Oslo, Norway). Cells were washed twice with phosphate-buffered saline, counted, and resuspended at the desired concentration.

Establishment of lymphoblastoid cell lines was performed in our laboratory by infecting peripheral blood lymphocytes with the Epstein–Barr virus as described previously [31]. Cells were grown in suspension in T flasks in an upright position, in approximately 10 ml RPMI 1640 (Gibco, BRL San Francisco, CA, USA) medium that contained 2 mM l-glutamine, 100 μg/ml penicillin/streptomycin and, unless otherwise stated, 10% (v/v) fetal bovine serum, and was maintained in a humidified 5% carbon dioxide incubator at 37°C. Fluid was routinely changed every 2 days by removing the medium above the settled cells and replacing it with an equal volume of fresh medium.

### Cell extracts

To prepare cell extracts, cells were harvested, washed in phosphate-buffered saline and then lysed in ice-cold lysis buffer (50 mM Tris pH 7.4, 150 mM NaCl, 50 mM NaF, 1% Nonidet P-40) containing 1 mM sodium orthovanadate, 1 mM phenylmethylsulfonyl fluoride, 1 mM sodium pyrophosphate and protease inhibitor Complete Mini Mixture (Roche, Mannhein, Germany). When preparing cell extracts from PBMCs, cells were pretreated with red blood cell lysis buffer (154 mM NH_4_Cl, 14 mM NaHCO_3_, 0.1 mM ethylenediamine tetraacetic acid) for 5 minutes, to remove red blood cells from the PBMC pellet. The protein content of the extracts was determined by the BCA protein assay kit (Thermo Scientific Alcobendas, Madrid, Spain).

### Western blot analysis

Protein (40 μg) from cell extracts were fractionated on a SDS polyacrylamide gel and transferred to a polyvinylidene fluoride membrane, which was then blocked with 5% bovine serum albumin and incubated overnight at 4°C, with primary antibodies at the following dilutions: 1:500 anti-CaM (FL-149; Santa Cruz Biotechnologies, Santa Cruz, CA, USA) and 1:5,000 anti-β-actin (Sigma Aldrich, Alcobendas, Madrid, Spain). Signals from the primary antibodies were amplified using species-specific antisera conjugated with horseradish peroxidase (Bio-Rad Richmond, CA, USA) and were detected with a chemiluminescent substrate detection system (ECL; Amersham, Uppsala, Sweden). The specificity of the antibodies used in this work was checked by omitting the primary antibodies in the incubation medium. Protein band densities were quantified using Image J software (NIH, Bethesda, MD, USA) after scanning the images with a GS-800 densitometer from Bio-Rad. To compare the results between experiments, all results were normalized by a standard sample included in every western blot. CaM levels of each individual were analyzed at least in two different experiments.

### Mass spectrometry analysis of calmodulin

For CaM identification, 40 μg protein from cell lysates were loaded in SDS-PAGE. The gel was then stained with SYPRO®Ruby (Invitrogen, Carlsbad, CA, USA), and the band of interest was cut, destained and washed, and after dithiothreitol reduction and iodoacetamide alkylation was digested with trypsin. Peptides were extracted from the gel and then analyzed in an LTQ Orbitrap Velos (Thermo-Scientific, Alcobendas, Madrid, Spain) coupled to a nanoEasy HPLC (Proxeon, Odense, Denmark). Peptides were first trapped onto a C18-A1 ASY-Column 2 cm precolumn (Thermo-Scientific, Alcobendas, Madrid, Spain), and then eluted onto a Biosphere C18 column (C18, inner diameter 75 μm, 15 cm long, 3 μm particle size; NanoSeparations, Nieuwkoop, The Netherlands) and separated using a 80-minute gradient from 3 to 35% Buffer B (Buffer A, 0.1% formic acid/2% acetonitrile; Buffer B, 0.1% formic acid in acetonitrile) at a flow rate of 250 nl/minute. Mass spectra were acquired in the positive ion mode and in a data-dependent manner selecting the 15 most intense ions for fragmentation using collision-induced dissociation. Full scan spectra (m/z 300 to 1,600) were acquired in the Orbitrap with a target value of 1,000,000 at a resolution of 30,000 (at m/z 400) and MS2 spectra were acquired in the linear ion trap with a target value of 10,000 and normalized collision energy of 38%. Precursor ion charge state screening and monoisotopic precursor selection were enabled. Singly charged ions and unassigned charge states were rejected. Dynamic exclusion was enabled with a repeat count of 1 and exclusion duration of 30 seconds.

Acquired spectra were searched against the human SwissProt database (091813) using the Sequest search engine through PD1.4 (Thermo-Scientific, Alcobendas, Madrid, Spain). As for the search parameters, the precursor and fragment mass tolerance were set to 10 ppm and 0.8 Da, respectively. Carbamidomethylation of cysteines was set as a fixed modification, and oxidation of methionines was set as a dynamic modification. Two missed cleavages were allowed. Identified peptides were validated using the Percolator algorithm with a *q*-value threshold of 0.01.

### Enzyme-linked immunosorbent assay analysis

CaM levels were detected in plasma using a Human Calmodulin Elisa kit from MyBiosource (San Diego, CA, USA) according to the manufacturer’s instructions. Then 50 μl plasma were added in duplicate to the microtiter wells. Sensitivity of the assay was 1.0 pg/ml. The coefficient of variation in the same lot was <9%, and in a different lot was <10%. Spike recovery was 94 to 103%. Linearity was in the following ranges: 1:2 (96 to 101%), 1:4 (93 to 107%), 1:8 (92 to 100%), and 1:16 (96 to 108%).

### Statistical analysis

Normality was checked with the Kolmogorov–Smirnov test with a Dallal Wilkinson–Lilliefor *p* value. *p* >0.10 was observed, indicating that the data were consistent with a Gaussian distribution. Parametric tests were therefore used in the statistical analysis.

Statistical significance was estimated by one-way analysis of variance followed by the Tukey’s test for multiple comparisons. When appropriate, two-way analysis of variance or Student’s *t* test was performed. Differences were considered significant at *p* <0.05.

Receiver operating curves (ROC) were drawn by plotting the sensitivity against 1 – specificity for different cutoff values. Areas under the ROC curves were calculated. The best cutoff value to distinguish AD patients from controls or other diseases was selected to achieve a sensitivity and a specificity >80%, as described previously [32]. Sensitivity, specificity, and accuracy were calculated for that certain cutoff point. Statistical analyses were performed using GraphPad Prism 6.0 for Mac (San Diego, CA, USA).

## Results

### Calmodulin content in Epstein–Barr virus-immortalized lymphocytes from controls and individuals suffering from neurodegenerative disorders

The clinical characterization and demographic data of the study groups of controls and AD, FTD, DLB, PD, ALS and PSP patients are summarized in the upper part of Table 1. Lymphoblastoid cell lines were generated by transformation with the Epstein–Barr virus, and the CaM content was determined by western blotting. To confirm the identity of the immunosignal detected by western blotting as CaM, the band was analyzed by mass spectrometry as described in Methods. Five peptides were identified with high confidence (Additional file 1), confirming that the band detected by western blotting indeed corresponded with CaM.

**Table 1:** Summary of the study population

	**Number**	**Age**		**Gender**	
		**Mean**	**Age range**	**Male**	**Female**
**Lymphoblasts**					
HC	33	64 ± 13	(31 to 83)	12	21
MCI	8	73 ± 6	(62 to 82)	4	4
AD					
Total	35	75 ± 7	(59 to 90)	16	19
Mild	18	76 ± 6	(60 to 83)	8	10
Moderate	10	76 ± 7	(59 to 84)	4	6
Severe	7	73 ± 10	(60 to 90)	4	3
FTD	7	64 ± 6	(54 to 70)	0	7
DLB	4	73 ± 15	(58 to 91)	3	1
PD	20	69 ± 10	(49 to 83)	14	6
ALS	10	63 ± 10	(47 to 79)	6	4
PSP	5	79 ± 6	(72 to 85)	1	4
**Peripheral blood mononuclear cells**					
HC	15	68 ± 8.3	(57 to 81)	4	11
MCI	7	68 ± 7.6	(55 to 76)	4	3
AD					
Total	21	77 ± 9	(58 to 88)	9	12
Mild	10	73 ± 9	(60 to 85)	5	5
Moderate	10	79 ± 8	(58 to 86)	4	6
Severe	1	88	88	0	1

Values expressed as mean ± standard deviation. HC, healthy control individuals, no sign of neurological disease; MCI, patients with a diagnosis of mild cognitive impairment; AD, patients with a diagnosis of probable Alzheimer’s disease; FTD, frontotemporal dementia individuals carrying a loss-of-function *PGRN* mutation; DLB, patients diagnosed for dementia with Lewy bodies; PD, patients with probable Parkinson’s disease; ALS, patients with a diagnosis of amyotrophic lateral sclerosis; PSP, patients diagnosed of progressive supranuclear palsy.

**Figure 1:**
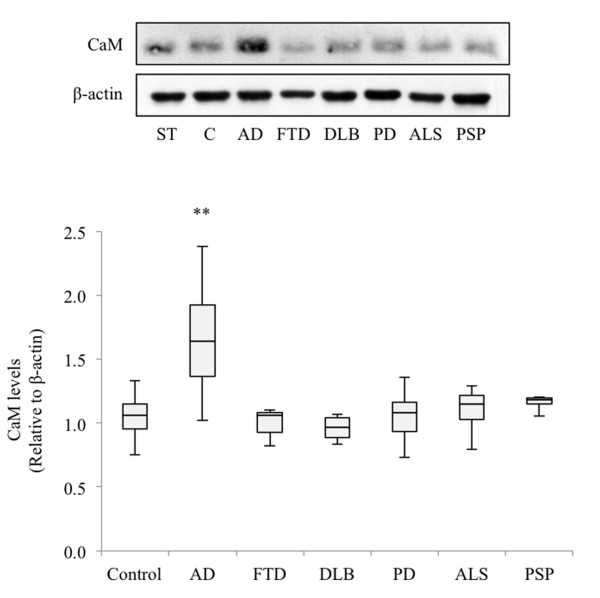
**Calmodulin levels in lymphoblasts from control, Alzheimer’s disease and other neurodegenerative disease patients.** Immortalized lymphocytes from all of the individuals listed in the upper part of Table 1 were seeded at an initial density of 1 × 10^6^/ml and cultured for 24 hours in RPMI medium containing 10% fetal bovine serum. At that time, cells were harvested and calmodulin (CaM) was detected by immunoblotting. A representative experiment is shown. Band intensity was measured and normalized by that of β-actin. To compare the results between experiments, the same standard sample (ST) was included in every western blot assay, and all the values were referred to ST CaM levels. At least two different experiments were performed with each individual. Box plots represent the CaM content in lymphoblasts from healthy and neurodegenerative disease patients. (**p <0.0001, significantly different from control and the other neurodegenerative diseases). C, control; AD, Alzheimer’s disease; FTD, frontotemporal dementia; DLB, dementia with Lewy bodies; PD, Parkinson’s disease; ALS, amyotrophic lateral sclerosis; PSP, progressive supranuclear palsy.

The cellular levels of CaM in lymphoblasts from individuals of the above study groups are presented in Figure 1. We found that CaM levels were significantly higher in AD subjects than in controls. In contrast, we did not find differences between cells from controls or patients affected by other forms of dementia, such as DLB, or FTD associated with a loss-of-function *PGRN* mutation (c-709-1G > A), as well as cells from patients suffering from other neurodegenerative disorders such as PD, ALS or PSP.

**Figure 2:**
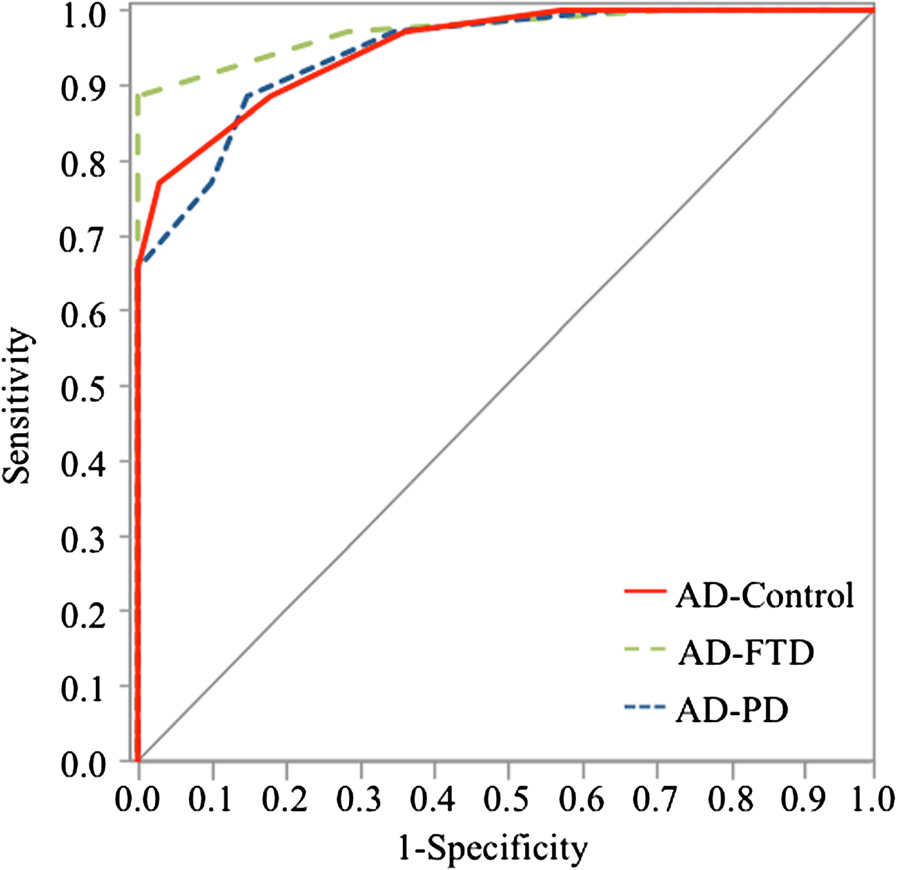
**Receiver operating characteristic curve analysis comparing Alzheimer’s disease patients and control, frontotemporal dementia or Parkinson’s disease patients.** Receiver operating characteristic (ROC) curve analysis of differentiation between Alzheimer’s disease (AD) patients and healthy controls, between AD and frontotemporal dementia (FTD) patients, and between AD and Parkinson’s disease (PD) patients. The area under the ROC curve (AUC) was 0.945 for AD versus control, 0.979 for AD versus FTD, and 0.944 for AD versus PD.

To evaluate the diagnostic performance of CaM content as an AD biomarker, ROC curves were generated (Figure 2). AD was compared with control, FTD and PD patients. The areas under the ROC curves were 0.945 (*p* <0.0001) for the classification control/AD, and 0.979 (*p* <0.0001) and 0.944 (*p* <0.0001) for AD/FTD and AD/PD respectively. Sensitivity, specificity and accuracy were calculated for the optimal cutoff point to distinguish AD patients and were the following: 0.886/0.818/0.853 for control/AD; 0.886/1/0.905 for AD/FTD; and 0.886/0.85/0.873 for AD/PD.

### Calmodulin content, age, gender and Alzheimer’s disease severity

**Figure 3:**
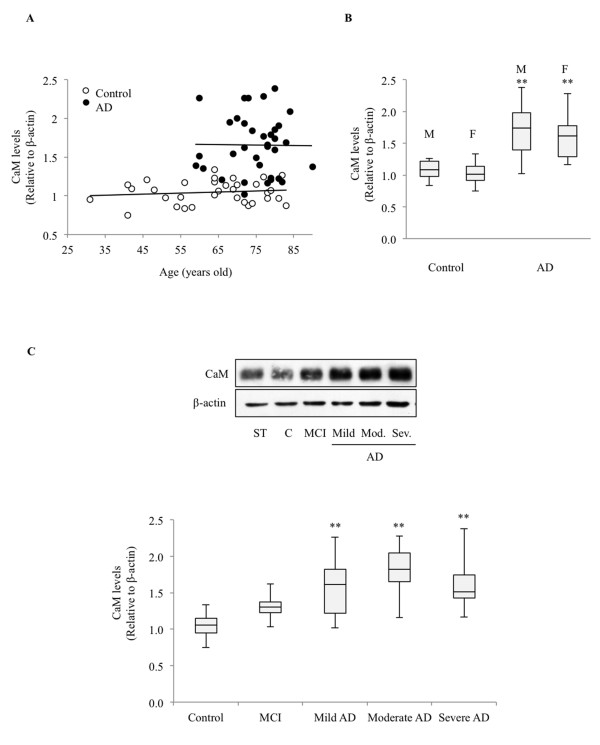
**Influence of age, gender and disease severity on calmodulin levels. (A)** Scatter plot of calmodulin (CaM) levels versus age in Alzheimer’s disease (AD) patients and controls. Correlation lines related to control and AD distributions are indicated. **(B)** Box plot of CaM levels in male (M) and female (F) controls and AD patients. (**p <0.0001, significantly different from both male and female controls). **(C)** Box plot of CaM levels of control, mild cognitive impairment (MCI) patients and AD patients classified within severity segments, as described in the upper part of Table 1. (**p < 0.0001, significantly different from control).

To better characterize the difference in CaM content of control and AD lymphoblasts, we studied whether this feature correlated with parameters associated with AD such as age or disease severity. AD patients were classified in three groups according to Diagnostic and Statistical Manual of Mental Disorders III-R criteria: mild (Mini-Mental State Examination score between 18 and 24), moderate (Mini-Mental State Examination score between 10 and 18), and severe (Mini-Mental State Examination score <10). A group of patients with amnesic MCI, which could constitute a prodromal stage of AD because these patients have a high risk of progression to AD [33], was also included. CaM content in both control and AD patients appears to be independent of age, as the difference between the slopes was not significant (*p* = 0.80) (Figure 3A). Likewise, the effect of gender was considered not significant (*p* = 0.70) (Figure 3B). The CaM content in AD patients was also independent of disease severity as there were no differences between lymphoblasts from early or advanced disease patients (Figure 3C). CaM levels were increased even in the MCI group, although to a lesser extent than in the AD patients (*p* = 0.06 MCI relative to control individuals, *p* = 0.26 when comparing MCI and mild AD patients).

### Calmodulin content in PBMCs from control and Alzheimer’s disease subjects

**Figure 4:**
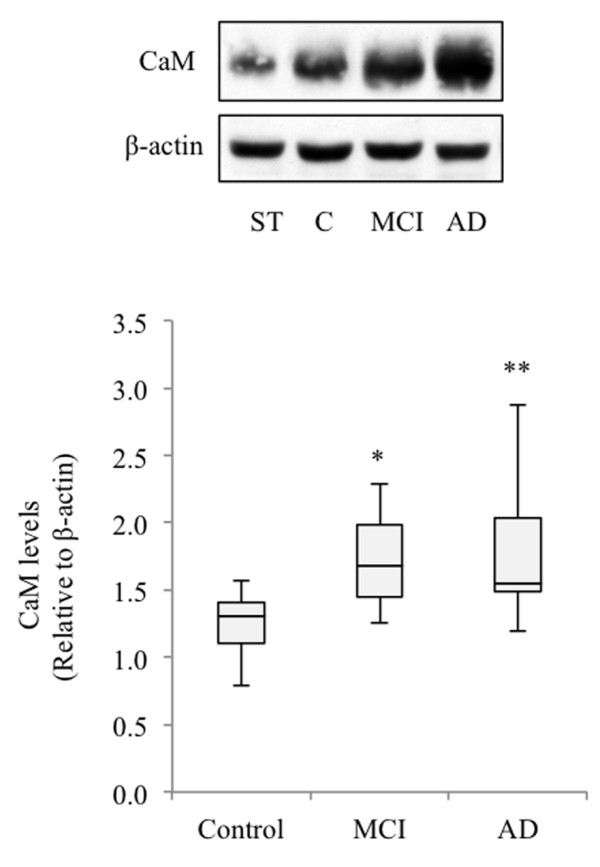
**Calmodulin content in freshly isolated peripheral blood mononuclear cells from control, mild cognitive impairment and Alzheimer’s disease patients.** Peripheral blood mononuclear cells were isolated from freshly obtained blood from 15 control individuals, seven mild cognitive impairment (MCI) patients and 21 Alzheimer’s disease (AD) patients (Table 1, lower part) following density gradient centrifugation as described in Methods. Cells were washed twice in phosphate-buffered saline, lysed in ice-cold lysis buffer and subjected to immunoblot. A representative experiment is shown. Band intensity was measured and normalized by that of β-actin. To compare the results between experiments, the same standard sample (ST) was included in every western blot, and all values were referred to ST CaM levels. At least two different experiments were performed with each individual. Box plots represent CaM content in lymphoblasts from healthy, MCI and AD patients. (*p <0.05, **p <0.01 significantly different from control).

Considering that immortalized lymphocytes are proliferating cells and that CaM has long been implicated in cell cycle regulation [34,35], we were interested in verifying whether differences in CaM levels are also evident between freshly isolated quiescent PBMCs from control and AD patients. Moreover, PBMCs, rather than immortalized lymphocytes, are considered convenient material for molecular diagnosis in clinical practice. For these experiments, we collected samples from 15 control individuals, seven MCI patients and 21 AD patients (see Table 1, lower part). Approximately 5 × 10^6^ cells derived from control or AD individuals were taken to prepare cell extracts. Figure 4 shows the results of the densitometric analyses of the corresponding immunoblots, revealing higher CaM content in quiescent mononuclear cells from MCI and AD patients when compared with control cells, thus indicating that the regulation of CaM content is not affected by the viral transformation.

### Analysis of calmodulin content in plasma by enzyme-linked immunosorbent assay

**Figure 5:**
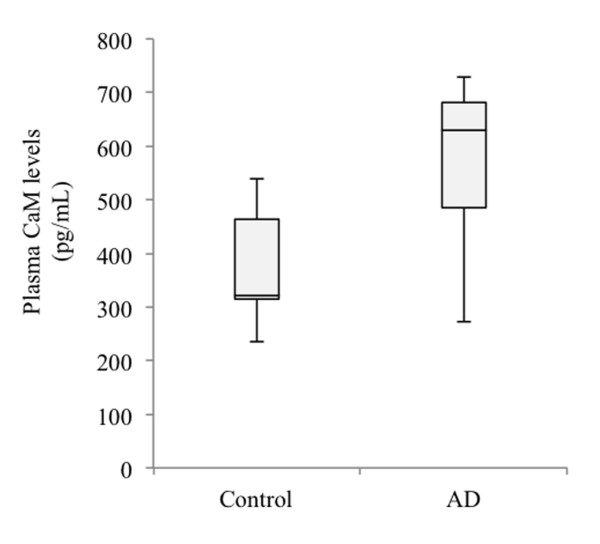
**Calmodulin levels in plasma from control and Alzheimer’s disease patients.** Box plots represent the calmodulin (CaM) concentration in plasma samples from healthy controls and Alzheimer’s disease (AD) patients. Plasma was obtained after blood centrifugation from six control individuals and six AD patients, and CaM levels were measured by enzyme-linked immunosorbent assay. (*p* = 0.09, two-tailed, unpaired Student’s *t* test).

Considering the need for a suitable assay for diagnostic purposes, easy to perform in already existing clinical infrastructures for analyses of blood, we were interested in evaluating whether quantitatively determined CaM levels in plasma by enzyme-linked immunosorbent assay would also serve to identify AD patients. A commercially available CaM enzyme-linked immunoassay was used for these experiments (MyBiosource, San Diego, CA, USA). Figure 5 shows that in AD patients the circulating CaM levels tended to be higher than in the control group (median (interquartile interval) 321 (315 to 464) vs. 629 (485to 682); *P* = 0.09), suggesting that measurement of CaM in plasma could help in AD diagnosis. Further work is needed to replicate this finding in larger and independent populations of patients.

## Discussion

Current therapies and treatments for AD are only symptomatic and the disease remains relentlessly progressive. Thus, our need to fully understand the pathogenesis of AD and to design molecular diagnostics and improved pharmacotherapies is vitally important to the healthcare system. The aim of the present work was to evaluate whether the content of CaM in easily accessible, peripheral cells could support the clinical diagnosis and discriminate AD from other causes of dementia or even between different stages of the disorder.

We previously reported increased CaM content in lymphoblasts from AD patients, as a consequence of impaired proteasomal degradation of the protein [19]. The higher CaM content was associated with impaired cell survival/death mechanisms [15,36]. Since cell cycle dysfunction seems to be a convergent point in neurodegenerative diseases [37], it was interesting to determine whether the control failure of CaM levels was commonly involved in neurodegenerative processes or, otherwise, was a disease-specific marker.

We first checked whether freshly isolated lymphocytes from AD patients also show increased levels of CaM as AD lymphoblasts do [19]. Our results indicate that the regulation of CaM content is not affected by the viral transformation, because higher levels of CaM were indeed found in PBMCs from AD patients than in control cells. This observation highlights the usefulness of Epstein–Barr virus-transformed lymphocytes as an experimental model.

Our results indicate that increased CaM content is a distinct feature of AD, since it was not observed in cells derived from patients with FTD, carriers of the *PGRN* mutation c.709-1G > A, DLB, PD, ALS or PSP even considering that cell cycle disturbances had been also described in cells from patients of FTD with *PGRN* mutations [38] and in other neurodegenerative diseases [37]. What seems to be unique for AD is the cell sensitivity to CaM-mediated cell survival control, secondary to increased levels of CaM.

Interestingly, no alteration in CaM levels was found in DLB, despite the fact that this dementia shares clinical and pathological characteristics with AD [39].

The changes in CaM content found in AD lymphocytes did not correlate with disease severity, suggesting that this is an early manifestation of the disease. This observation is in agreement with cell cycle theory, which states that cell cycle-related event-induced neurodegeneration is not the result of accumulated loss of neurons but rather an early feature in the instigation of the disease [40]. This lack of correlation would also suggest that the cellular CaM content is a trait disease marker, not reflecting the disease status. Accordingly, we have also detected increased levels of CaM in MCI individuals, suggesting the potential of this variable in differentiating MCI and asymptomatic individuals.

ROC curve analyses indicate that the CaM content in peripheral cells is specific and sensitive enough for AD diagnosis. For the control/AD classification, the area under the ROC curve was 0.945 (95% confidence interval 0.896, 0.994) with positive or negative predicted values of 89% and 88% respectively. The overall diagnostic accuracy was 0.853, similar to currently accepted cerebrospinal fluid biomarkers (tau, phospho-tau amyloid-beta 1–42) [41]. In addition, determination of the CaM content in peripheral cells also has the potential of discriminating AD patients from subjects affected by other forms of dementia as well as from other neurodegenerative diseases. Taken together, our results add further support to the usefulness of peripheral lymphocytes for the search of convenient biomarkers for AD [42-44].

Interestingly, a trend towards higher plasma circulating levels of CaM in AD subjects versus nondemented individuals was detected by enzyme-linked immunosorbent assay, a more convenient assay in clinical practice. However, further work is needed with a larger number of control and patients affected by AD and other neurodegenerative disorders.

AD pathogenesis is very complex. According to the cell cycle hypothesis, a dysfunction in the G_1_/S checkpoint could play a role in instigation of the disease [5,45,46]. We previously reported the existence of a molecular link between decreased levels of the CDK inhibitor p27, and increased phosphorylation of pRb protein and proliferation of AD lymphoblasts [12]. On the other hand, the CaM content appears to regulate the rate of p27 degradation in AD cells through a phosphoinositide-3 kinase/Akt-dependent mechanism [15]. Moreover, higher levels of CaM also correlated with the resistance of AD cells to serum deprivation-induced apoptosis [36]. CaM thus seems to play a pivotal role in transmitting proliferative/survival signals from the plasma membrane to the nucleus. Whether CaM contributes to cell proliferation or apoptosis depends on cellular CaM levels as well as the presence of growth-stimulatory signals. To our knowledge, there is very little information about CaM levels in AD brain; however, impaired CaM-dependent activation of CaMKII or phosphoinositide-3 kinase/Akt has also been described in AD brain [47,48], and therefore it is tempting to speculate that changes in CaM levels in AD lymphoblasts may be another peripheral sign of the disease. Altered CaM levels in AD brain could play a role in the cell cycle disturbance-induced neuronal apoptosis.

In summary, our study reveals significant changes in CaM levels in peripheral cells from AD patients and MCI individuals. Our findings indicate that peripheral cell CaM content has potential diagnostic power, differentiating AD from other types of dementia, as well as from other neurodegenerative disorders. Nevertheless, further work with larger and independent populations of patients will be needed before altered CaM content can be considered a suitable biomarker for AD diagnosis.

## Conclusion

CaM levels measured in peripheral cells could be considered a useful biomarker to help in early diagnosis of AD, enabling one to discriminate AD from other dementias with high levels of sensitivity and specificity.

### Note

This article is part of a series on *Peripheral Biomarkers*, edited by Douglas Galasko. Other articles in this series can be found at http://alzres.com/series/biomarkers.

## Abbreviations

AD: Alzheimer’s disease; ALS: Amyotrophic lateral sclerosis; CaM: Calmodulin; DLB: Dementia with Lewy bodies; FTD: Frontotemporal dementia; MCI: Mild cognitive impairment; PBMC: Peripheral blood mononuclear cell; PD: Parkinson’s disease; PGRN: Progranulin; PSP: Progressive supranuclear palsy; ROC: Receiver operating characteristic.

## Competing interest

The authors declare that they have no competing interests.

## Authors’ contributions

NE and AM-R conceived and designed the experiments. NE, CA and AdlE carried out the experimental work. NE analyzed the data, prepared the figures and performed the statistical analysis. AV and FB-P recruited and diagnosed the patients and provided the blood samples. AM-R and NE wrote the manuscript. All authors read and approved the final manuscript.

## Supplementary Material

Additional file 1: Table S1 Presenting identification of CaM by mass spectrometry. Click here for file
