# Supplementary material for: Calmodulin levels in blood cells as a potential biomarker of Alzheimer’s disease
Source: Alzheimers Res Ther. 2013 Nov 7;5(6):55. doi: 10.1186/alzrt219 (PMC3978675; doi:10.1186/alzrt219)
Supplement: Additional file 1: Table S1 — Presenting identification of CaM by mass spectrometry. [file alzrt219-S1.docx]

| **Accession** | **Description** | **Score** | **Coverage** | **# Unique Peptides** | **# Peptides** |  |  |  |
| --- | --- | --- | --- | --- | --- | --- | --- | --- |
| P62158 | **Calmodulin OS=Homo sapiens GN=CALM1 PE=1 SV=2 - [CALM_HUMAN]** | 46.91 | 44.30 | 5 | 5 |  | | |
| Peptides | **Confidence level** | **Sequence** | **Missed cleavages** | **Charge** | **q-Value** | **XCorr** | **m/z (Da)** | **MH+ (Da)** |
|  | High | EADIDGDGQVNYEEFVQmmTAK | 0 | 3 | 0 | 4.89 | 841.36200 | 2522.07145 |
|  | High | EADIDGDGQVNYEEFVQmmTAK | 0 | 3 | 0 | 4.85 | 841.36304 | 2522.07456 |
|  | High | EADIDGDGQVNYEEFVQmmTAK | 0 | 2 | 0 | 4.21 | 1261.53906 | 2522.07085 |
|  | High | EAFSLFDKDGDGTITTK | 1 | 2 | 0 | 4.16 | 922.94928 | 1844.89128 |
|  | High | VFDKDGNGYISAAELR | 1 | 2 | 0 | 3.97 | 877.93866 | 1754.87004 |
|  | High | EADIDGDGQVNYEEFVQmmTAK | 0 | 2 | 0 | 3.91 | 1261.54053 | 2522.07378 |
|  | High | EADIDGDGQVNYEEFVQmmTAK | 0 | 2 | 0 | 3.87 | 1261.53870 | 2522.07012 |
|  | High | VFDKDGNGYISAAELR | 1 | 3 | 0 | 3.79 | 585.62897 | 1754.87235 |
|  | High | EAFSLFDKDGDGTITTK | 1 | 2 | 0 | 3.54 | 922.94666 | 1844.88603 |
|  | High | EAFSLFDKDGDGTITTK | 1 | 3 | 0 | 2.95 | 615.63556 | 1844.89212 |
|  | High | mKDTDSEEEIR | 1 | 3 | 0 | 2.58 | 456.87048 | 1368.59690 |
|  | High | mKDTDSEEEIR | 1 | 2 | 0.002 | 2.17 | 684.80011 | 1368.59294 |
|  | High | DGNGYISAAELR | 0 | 2 | 0.003 | 2.02 | 633.30920 | 1265.61113 |

**SUPPLEMENTARY TABLE 1.** Identification of Calmodulin by Mass Spectrometry.
